# A Case of Post-vomiting Pneumorachis With Cardiac Arrest: Boerhaave or Macklin?

**DOI:** 10.7759/cureus.85905

**Published:** 2025-06-13

**Authors:** Jérôme A Dehon

**Affiliations:** 1 Intensive Care Unit, CHU (Centres Hospitaliers Universitaires) Helora, Nivelles, BEL; 2 Intensive Care, Université Catholique de Louvain, Brussels, BEL

**Keywords:** barotrauma, epidural emphysema, esophageal perforation, macklin effect, pneumomediastinum, pneumorachis, roux-en-y gastric bypass, vomiting

## Abstract

Pneumorachis, defined as air within the spinal canal, is a rare radiological finding, typically associated with trauma or iatrogenic causes. Its spontaneous occurrence following vomiting raises important diagnostic concerns, notably the possibility of esophageal perforation or barotrauma via the Macklin effect.

We report a case of a 37-year-old man with a history of Roux-en-Y gastric bypass and chronic alcohol use, found collapsed at home after a possible 48-hour period on the floor. Shortly after initial mobilization by emergency responders, he developed sudden cardiac arrest, with return of spontaneous circulation achieved within two minutes. Following transport and hospital admission, he remained hypotensive and required ICU admission with mechanical ventilation. A whole-body CT scan, performed during the transfer from the emergency department to the intensive care unit, revealed extensive pneumorachis from the cervical to thoracic spine and associated pneumomediastinum without pneumothorax or free fluid. Neurological evaluation was initially not feasible due to sedation. Collateral history revealed repeated episodes of forceful vomiting prior to presentation. Upper endoscopy showed no obvious esophageal rupture. After sedation was weaned, neurological examination showed no focal deficits. A transient vertical nystagmus, likely due to thiamine deficiency in the context of previous gastric surgery and alcohol misuse, resolved with supplementation. The patient recovered fully under conservative management, with follow-up imaging showing complete resolution.

The findings were consistent with barotrauma-induced air dissection, most likely via the Macklin effect. However, a self-contained esophageal microperforation could not be completely ruled out. This case highlights the importance of structured evaluation and clinical restraint in the presence of dramatic imaging findings.

Spontaneous pneumorachis, though alarming on imaging, may follow a benign course once life-threatening causes have been excluded. Awareness of its rarity and potential diagnostic pitfalls is key to avoiding unnecessary invasive interventions.

## Introduction

Pneumorachis, the presence of air within the spinal canal, is a rare and often incidental radiological finding [1,2]. It is typically seen in the context of trauma or iatrogenic causes [3,4], and is only exceptionally observed following non-traumatic events. Spontaneous pneumorachis, particularly when accompanied by pneumomediastinum, may pose a significant diagnostic challenge [5].

Forceful vomiting can lead to barotrauma and alveolar rupture, with air dissecting through perivascular sheaths toward the mediastinum - a mechanism known as the Macklin effect [6]. Occasionally, the air can extend into the spinal canal through the neural foramina. However, in patients with vomiting and pneumomediastinum, a transmural esophageal rupture (Boerhaave syndrome) must first be excluded, given its potential severity and need for urgent intervention [7].

We report a case of a young man with a history of Roux-en-Y gastric bypass who developed extensive cervicothoracic pneumorachis and pneumomediastinum following repeated episodes of vomiting. The striking radiological findings initially raised concern for life-threatening pathology, yet the clinical course proved benign under conservative management. This case highlights the diagnostic complexity of such unusual presentations and the importance of interpreting striking imaging findings within the broader clinical context.

## Case presentation

A 37-year-old man with a history of Roux-en-Y gastric bypass and chronic alcohol use was brought to the emergency department after being found collapsed at home. He may have remained on the floor for up to 48 hours. Shortly after initial mobilization by emergency responders, he experienced a sudden cardiac arrest. Cardiopulmonary resuscitation was initiated immediately, and return of spontaneous circulation was achieved within two minutes following a single dose of intravenous epinephrine.

Despite fluid resuscitation, the patient remained hypotensive after admission to the emergency department and was transferred to the intensive care unit for vasopressor support and further evaluation. He was sedated, intubated, and mechanically ventilated, and his recent post-cardiac arrest status further limited the possibility of reliable neurological assessment.

**Figure 1:**
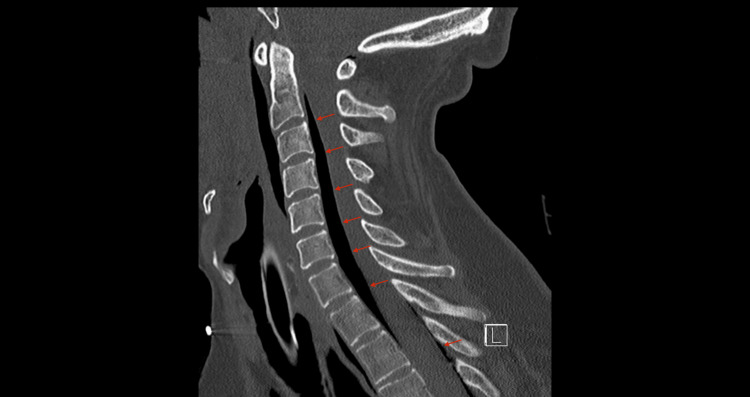
Sagittal CT reconstruction showing extensive cervicothoracic pneumorachis.

**Figure 2:**
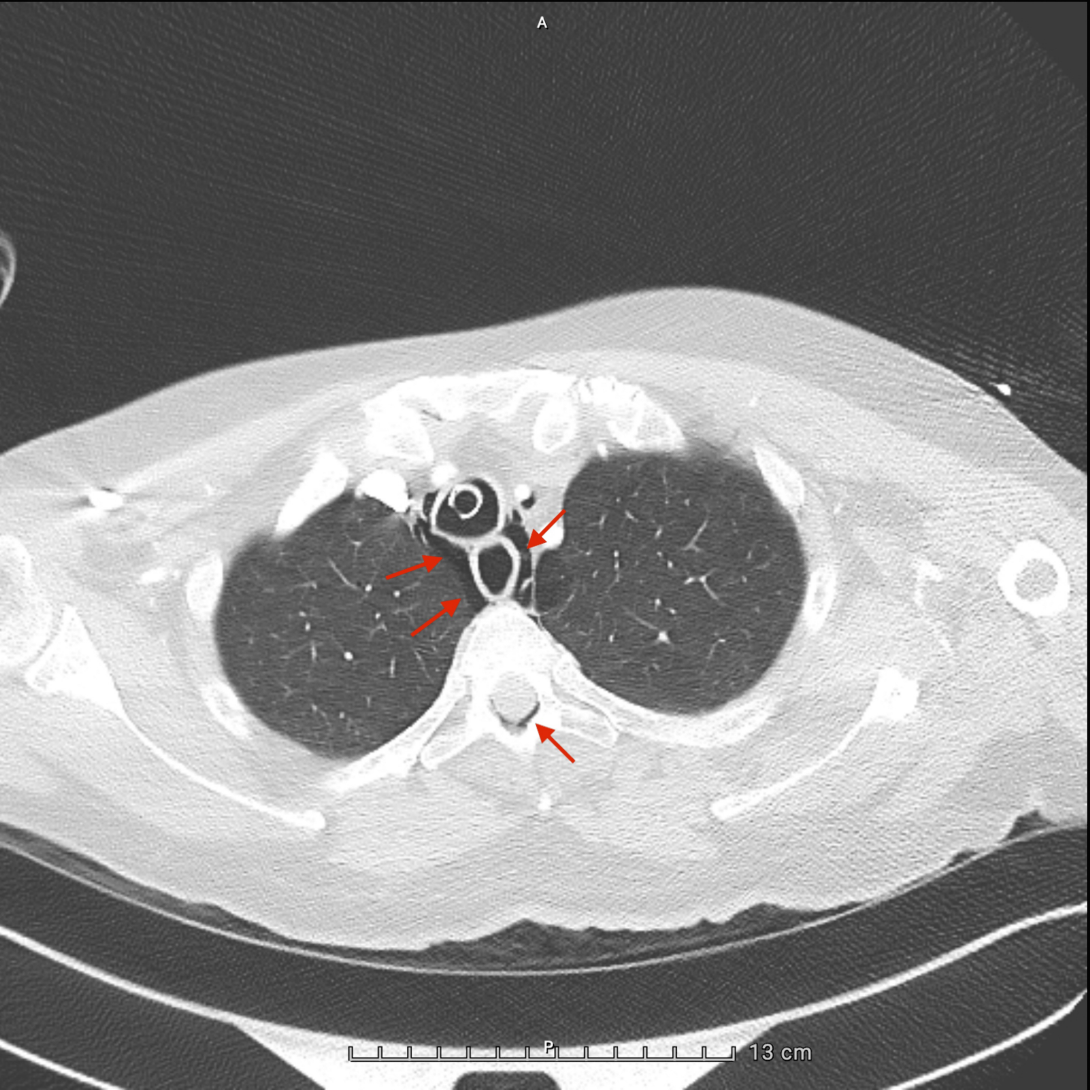
Axial chest CT – upper thoracic level slice showing pneumomediastinum and epidural air.

**Figure 3:**
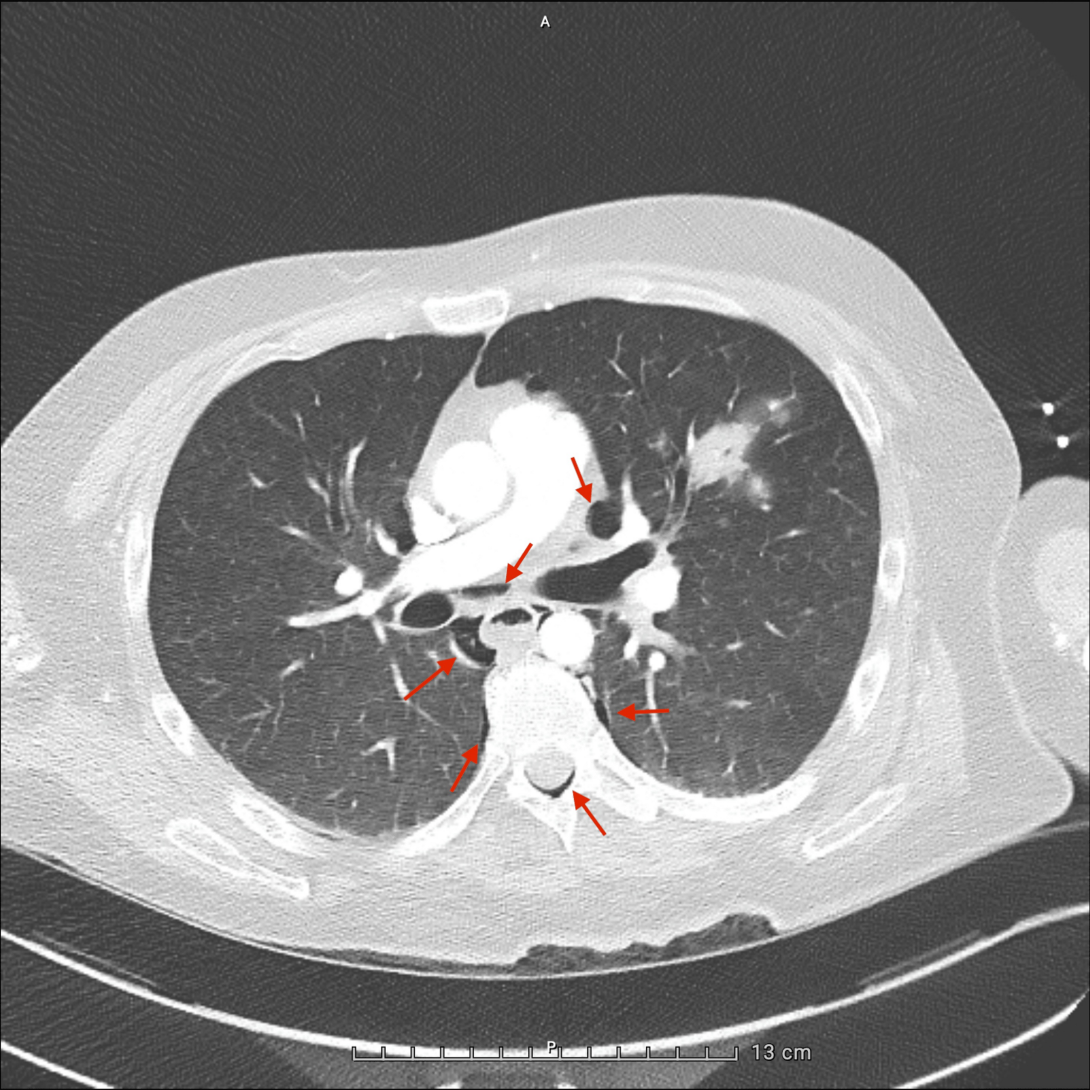
Axial chest CT – mid thoracic level slice showing pneumomediastinum and epidural air. A patchy consolidation is also noted in the left upper lobe, likely reflecting alveolar filling in the context of aspiration or prolonged immobilization.

**Figure 4:**
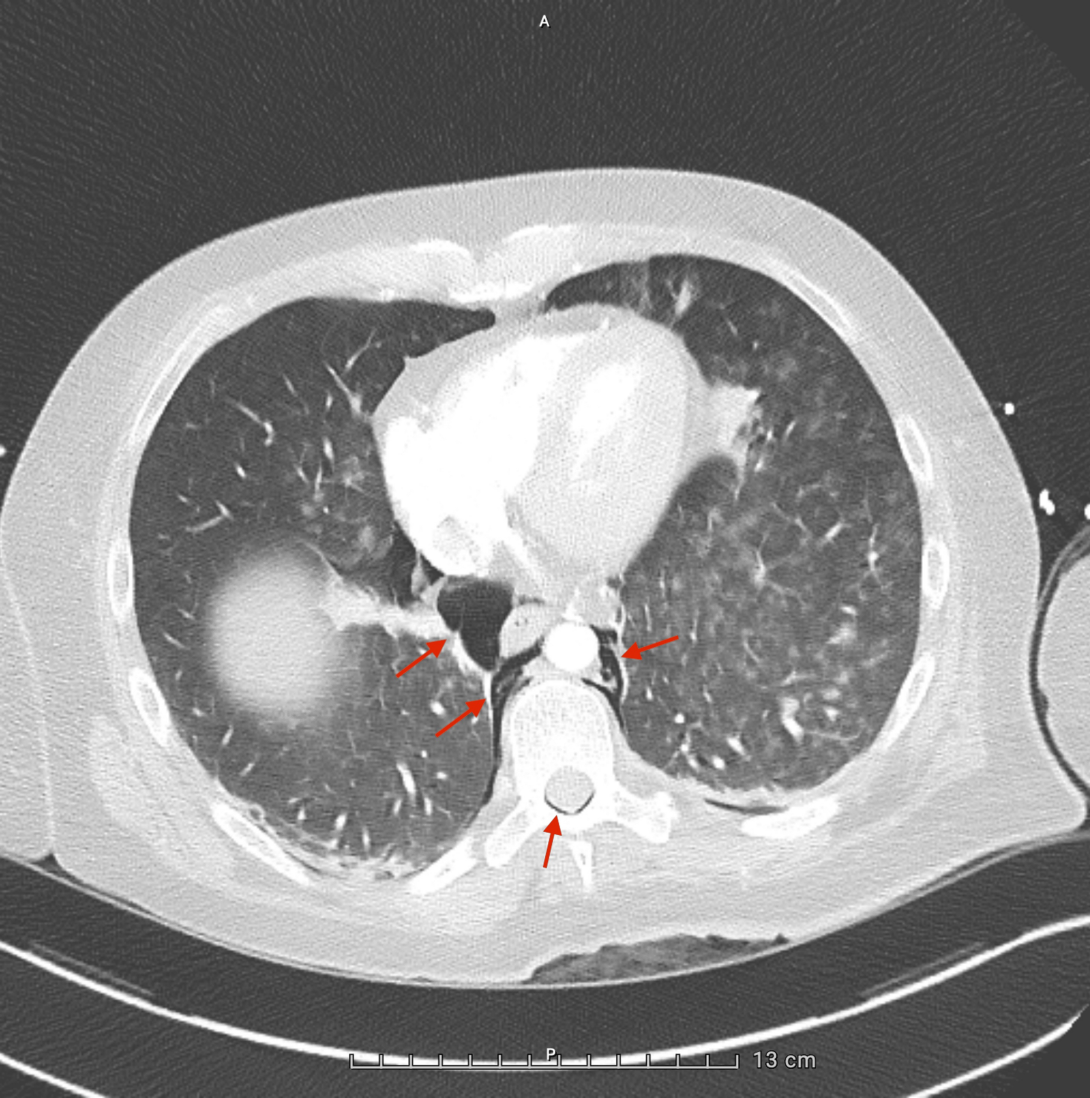
Axial chest CT – lower thoracic level slice showing pneumomediastinum and epidural air. Also showing multiple areas of ill-defined pulmonary opacities, predominantly in the left lung. These findings are consistent with bilateral alveolar filling, most likely of infectious origin.

A whole-body computed tomography (CT) scan, performed during the transfer from the emergency department to the ICU, revealed extensive air in the epidural space from the cervical to thoracic spine (Figure 1), along with pneumomediastinum surrounding the great vessels (Figures 2-4), in the absence of pneumothorax or free fluid. There were no signs of vertebral trauma or cranial air embolism.

Collateral history from the patient’s family revealed multiple episodes of intense vomiting in the days prior to presentation. Given the patient’s surgical history and the radiological findings, an esophageal perforation was initially suspected. An upper endoscopy was performed, but showed no evidence of a significant fistula or visible perforation.

After sedation was weaned, neurological examination revealed no focal deficits. A transient vertical nystagmus was observed and resolved following empirical thiamine supplementation. Although a brain MRI performed two weeks later showed no structural abnormalities suggestive of Wernicke’s encephalopathy, the clinical response supported this working diagnosis in the context of both prior bariatric surgery and chronic alcohol use.

The patient’s clinical status improved steadily with supportive care. Follow-up CT imaging showed complete resolution of both the pneumomediastinum and pneumorachis. The most likely etiology was alveolar barotrauma secondary to repeated vomiting, with air dissection into the mediastinum and epidural space.

## Discussion

**Figure 5:**
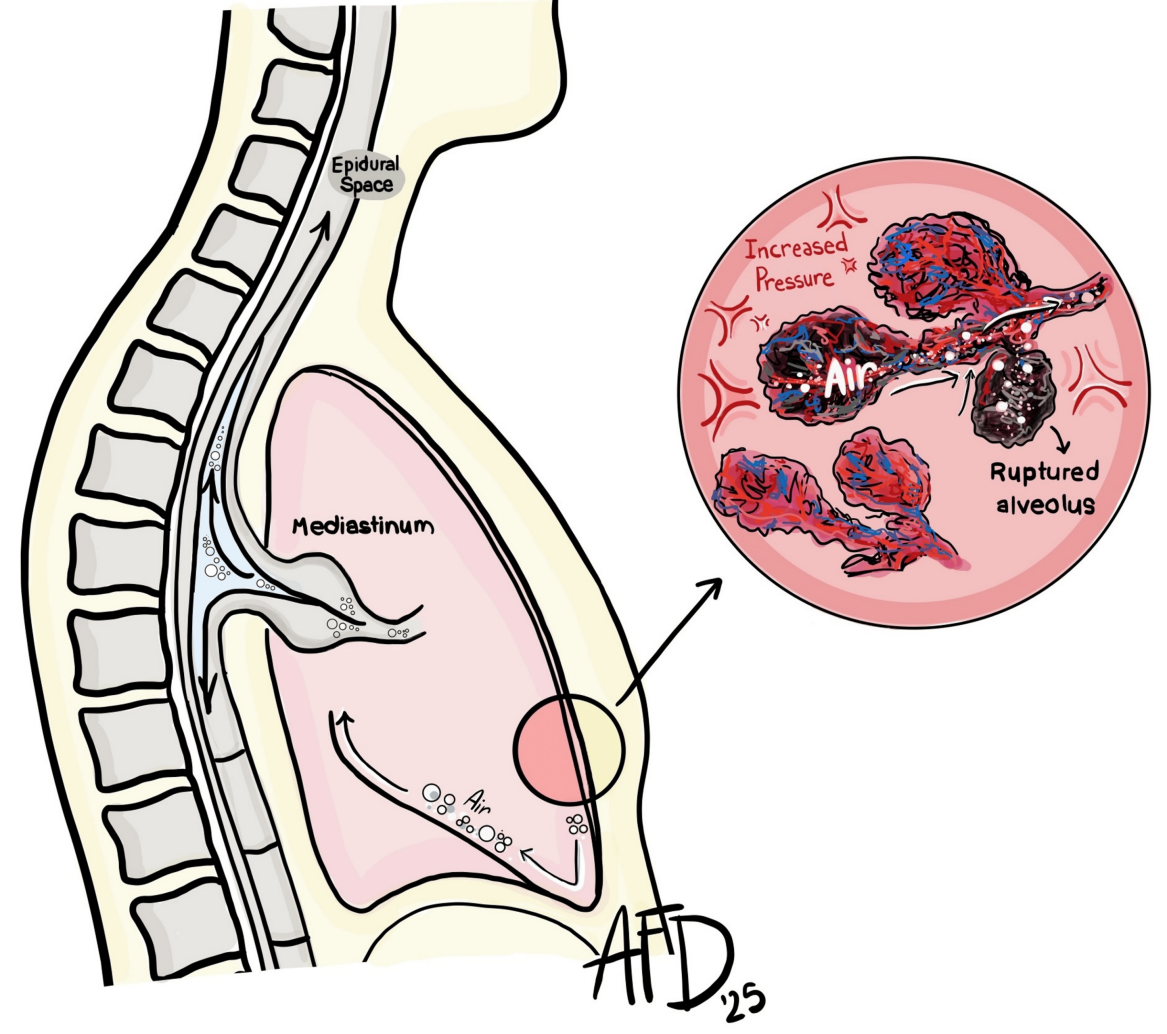
Schematic representation of the Macklin effect. Following alveolar rupture, air dissects along the bronchovascular sheaths toward the mediastinum, potentially tracking into the spinal canal via the neural foramina. Illustration by Arielle Fearn. Adapted from Grewal and Gillaspie [8].

Spontaneous pneumorachis (PR), defined as the presence of air in the spinal canal without trauma or recent instrumentation, is an exceptionally rare entity [1,3]. Most reported cases are linked to barotrauma-related pneumomediastinum, frequently attributed to the Macklin effect [5,6]. This mechanism involves alveolar rupture following a sudden rise in intra-alveolar pressure, with air dissecting along the bronchovascular sheaths toward the mediastinum, and potentially into the spinal canal via neural foramina (Figure 5) [6,8].

Although this physiopathological model provides a plausible explanation for our patient’s findings, the diagnostic process was not straightforward. The presence of vomiting, altered post-surgical anatomy, and pneumomediastinum also raised the concern for Boerhaave syndrome, a life-threatening esophageal rupture [7]. Urgent upper endoscopy did not reveal any obvious perforation, but a small, contained or spontaneously healed esophageal defect could not be definitively excluded.

This highlights a broader clinical challenge: spontaneous PR remains a diagnosis of exclusion. In patients with vomiting and pneumomediastinum, ruling out esophageal perforation is essential. This involves a combination of imaging, clinical monitoring, and endoscopic evaluation when indicated [7,8]. Grewal et al. notably recommend endoscopic assessment in cases of pneumomediastinum with emesis, especially when concerning features such as pneumorachis are present [8].

In our case, the initial neurological evaluation was limited due to sedation and post-arrest status. Once sedation was lifted, no focal neurological deficits or signs of spinal cord compression were observed. A transient vertical nystagmus, attributed to thiamine deficiency, resolved with supplementation, and no intracranial or spinal air was identified.

While our patient followed a benign clinical course, rare cases of symptomatic spinal cord compression due to epidural air have been documented. Ould-Slimane et al. and Oertel et al. each reported cases where pneumorachis caused neurological deficits requiring intervention [4,9]. Though uncommon, such reports emphasize the importance of careful neurological monitoring, particularly in sedated patients or those with unreliable examinations.

In the absence of neurologic symptoms or spinal cord compression, the presence of PR does not typically warrant intervention. Several case series have reported favorable outcomes with conservative management alone, relying on high-concentration oxygen therapy, monitoring, and treatment of the underlying cause [5,9,10]. Our case aligns with this approach, with complete clinical and radiologic resolution achieved through supportive care alone.

As more cases are reported, PR is increasingly understood as a dramatic but often benign radiological finding. This case adds to the growing literature suggesting that, once life-threatening causes such as esophageal rupture have been reasonably excluded, spontaneous pneumorachis can often be managed conservatively with good outcomes.

## Conclusions

Spontaneous pneumorachis is an uncommon and often striking radiological finding. When extensive, it may prompt significant concern and trigger aggressive diagnostic or therapeutic interventions. Yet, in the absence of neurological symptoms or overt signs of visceral perforation, it may represent a benign manifestation of barotrauma, particularly when associated with pneumomediastinum following vomiting. While the Macklin effect offers a plausible mechanism for air dissection into the epidural space, potentially life-threatening conditions such as esophageal rupture must always be actively excluded. This case reminds us that dramatic imaging should not overshadow clinical reasoning, and that management must remain rooted in structured, evidence-based assessment.
